# A rapid multiplex cell-free assay on biochip to evaluate functional aspects of double-strand break repair

**DOI:** 10.1038/s41598-022-23819-0

**Published:** 2022-11-21

**Authors:** Xavier Tatin, Giovanna Muggiolu, Sarah Libert, David Béal, Thierry Maillet, Jean Breton, Sylvie Sauvaigo

**Affiliations:** 1 LXRepair, 5 Avenue du Grand Sablon, 38700 La Tronche, France; 2 Univ. Grenoble Alpes, CEA, CNRS, IRIG, SyMMES, 38000 Grenoble, France

**Keywords:** Assay systems, DNA damage and repair, Double-strand DNA breaks

## Abstract

The repair of DNA double-strand breaks (DSBs) involves interdependent molecular pathways, of which the choice is crucial for a cell’s fate when facing a damage. Growing evidence points toward the fact that DSB repair capacities correlate with disease aggressiveness, treatment response and treatment-related toxicities in cancer. Scientific and medical communities need more easy-to-use and efficient tools to rapidly estimate DSB repair capacities from a tissue, enable routine-accessible treatment personalization, and hopefully, improve survival. Here, we propose a new functional biochip assay (NEXT-SPOT) that characterizes DSB repair-engaged cellular pathways and provides qualitative and quantitative information on the contribution of several pathways in less than 2 h, from 10 mg of cell lysates. We introduce the NEXT-SPOT technology, detail the molecular characterizations of different repair steps occurring on the biochip, and show examples of DSB repair profiling using three cancer cell lines treated or not with a DSB-inducer (doxorubicin) and/or a DNA repair inhibitor (RAD51 inhibitor; DNA-PK inhibitor; PARP inhibitor). Among others, we demonstrate that NEXT-SPOT can accurately detect decreased activities in strand invasion and end-joining mechanisms following DNA-PK or RAD51 inhibition in DNA-PK-proficient cell lines. This approach offers an all-in-one reliable strategy to consider DSB repair capacities as predictive biomarkers easily translatable to the clinic.

## Introduction

DNA double-strand breaks (DSBs) are considered the most deleterious type of DNA lesions. They are potent inducers of mutations and chromosomal aberrations, which may lead to hereditary conditions and, more frequently, to cancer. To maintain genomic stability, human cells need to rapidly initiate and orchestrate DSB repair by triggering a highly complex signaling network of molecular reactions, characterized by several repair pathways^1^.

Firstly, the canonical non-homologous end joining (NHEJ) pathway can be active all along the cell cycle and directly binds the two break ends without referring to any template. For these reasons, it is considered the predominant repair mechanism of all types of DSBs^2^. In contrast, homologous recombination (HR) is considered an error-free pathway and the optimal option for processing DNA DSBs during G2/S phase of the cell cycle, where a template material is available^3^. HR starts with the resection of DNA ends to form a single-stranded region that invades the homologous DNA regions. Then, several competing pathways complete the HR process, notably involving DNA synthesis^4,5^. Single-strand annealing (SSA) is sometimes characterized as a sub-pathway of HR due to its reliance on HR-related machinery and its dependence on homology, although it also involves deletions of genetic material and therefore, is deemed mutagenic^6^. Finally, an alternative end-joining pathway (alt-EJ, also named microhomology-mediated end joining, MMEJ) operates when NHEJ is disabled. It joins the two DNA ends in an error-prone way, as it often implies micro-deletions of the repaired sequences^2,7^. Not only HR, but also alt-EJ and SSA require resection of DNA ends to generate single-stranded DNA (ssDNA) tails of different sizes^5^. The length of ssDNA tails impacts the choice of the repair mechanism and it is followed by the annealing of 2–20 bp microhomologies for alt-EJ, more than 50 bp for SSA, and around 100 bp for HR^8^. DNA polymerases play a fundamental role in DSB repair as they can fill in the gaps to facilitate DNA strand ligation or add nucleotides to create homologies^9^. In practice, those pathways display complementary features with functional overlap and possible backup compensatory actions in case of inherited or acquired inability, i.e. in case of a loss-of-function genetic event or following pharmacological inhibition^10–12^.

In a study conducted by Xiao et al., it has been demonstrated that 11.8% of tumors harbor at least one genetic alteration in genes involved in DSB reapir^13^. The most commonly altered genes were *ATM* (19.1% of DNA repair-mutated samples), *BRCA2* (17.2%), *BRCA1* (10.9%), *RAD50* (8.9%), and *ATR* (7.8%). Defects in DDR capacities have been associated with high tumor mutational burden, microsatellite instability, and thus, enhanced response to immune checkpoint blockers^14,15^. DDR defects have also been exploited to develop targeted therapeutic strategies with DDR inhibitors as an approach to inhibit the compensatory pathway established by the tumor to bypass its DDR alteration (synthetic lethality)^16^. From another perspective, several germinal DDR defects and polymorphisms are linked either to severe side effect reactions following radiation therapy or to tumor radioresistance^17,18^.

Altogether, DDR defects are common in cancer and represent highly promising predictive biomarkers of response and/or toxicity to a wide range of anticancer treatments. Therefore, systematic diagnosis of DDR defects should be part of the ongoing strategy of care personalization to better identify the most optimal treatment modality for a particular individual. DDR capacities can be estimated by multiple methods, more or less sophisticated, at the gene, transcript, or protein levels^19,20^. However, no existing method measuring the functionality of DSBs repair meets all the criteria required for practical adoption in routine, neither for research nor for clinical applications. To this aim, we designed a breakthrough functional multiplex enzymatic assay (NEXT-SPOT), ease of use that characterizes specific steps of DSB repair pathways from cell lysates. Here, the different aspects of the method are presented. To illustrate the strength of this approach, we used the NEXT-SPOT assay to characterize DSB repair of three cancer cell lines at the basal level and following exposure to a DSB-inducing agent (doxorubicin), combined or not with different types of DNA repair inhibitors.

## Results

### NEXT-SPOT: a functional DSB repair fluorescent assay on a biochip

We designed an enzymatic cell-free DSB repair assay that simultaneously characterizes strand invasion, end-joining, and polymerase activities of the DSB repair machinery.

**Figure 1:**
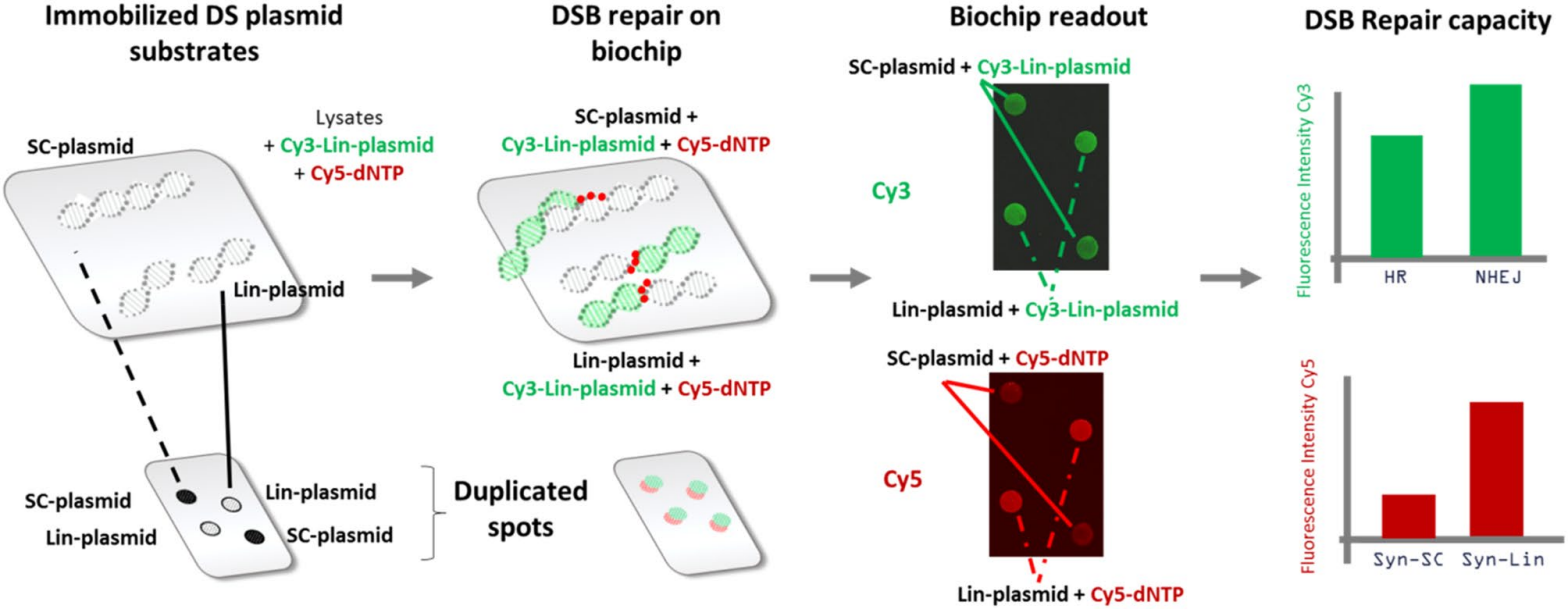
Overview of the principle of the NEXT-SPOT assay on biochip. Each biochip is composed of 14 pads, each pad contains two spots of two immobilized substrates: supercoiled plasmids (SC-plasmid) and linear plasmid (Lin-plasmid). Repair reactions occur on the support between the immobilized substrates and the Cy3-labelled linear plasmid (Cy3-Lin-plasmid), the biotin-dCTP, and the cell lysates added to each pad. Then, the biotin-labeled dCTP is revealed by Cy5-streptavidin. Alternatively Cy5-dCTP can be used. Signals are quantified using a dual-color laser-induced fluorescence scanner (excitation wavelengths: 532 and 635 nm). *SC-plasmid* supercoiled plasmid, *Lin-plasmid* linearized plasmid, *Syn-SC* DNA synthesis on SC-plasmid, *Syn-Li*: DNA synthesis on Lin-plasmid.

The assay quantifies the incorporation of different markers on two plasmid templates immobilized on biochip, the combination of which provides information on several DSB-specific repair steps (Fig. 1). The first immobilized supercoiled double-strand plasmid (SC-plasmid) serves predominantly as a substrate for an HR-like strand invasion. The second plasmid is immobilized in its linear form (Lin-plasmid) and serves preferentially as a substrate for DNA end-joining. The repair reaction occurs between the immobilized substrates, Cy3-labeled linearized plasmid (Cy3-Lin-plasmid), and labeled-dCTP provided in solution, in the presence of ATP, other dNTPs, by means of repair proteins contained in cell lysates. The nucleotides incorporation during the repair reactions gives insight into the synthesis activities in the tested samples and reflects the involvement of polymerases. In our system, a complementary strand is always available to allow the hybridization of this single strand, indeed in the presence of all reagents, the 3'-end can be used to start new DNA synthesis.

Consequently, the DNA repair profile of each sample is characterized by appraising four signals: (1) invasion of the Cy3-Lin-plasmid onto the immobilized SC-plasmid that mimics the initial step of strand invasion during HR; (2) ligation of the Cy3-Lin-plasmid onto the immobilized Lin-plasmid that represents the end-joining predominantly performed in cells by NHEJ; incorporation of biotin-dNTP (detected by Cy5-streptavidin) or of Cy5-dCTP (3) into the immobilized SC-plasmid, and (4) into the immobilized Lin-plasmid, which reveals the polymerase activities (named DNA Synthesis on supercoiled plasmid (Syn-SC) and DNA Synthesis on linear plasmid (Syn-Lin), respectively).

### Insight into the steps of the DSB repair reactions occurring on the biochip

**Figure 2:**
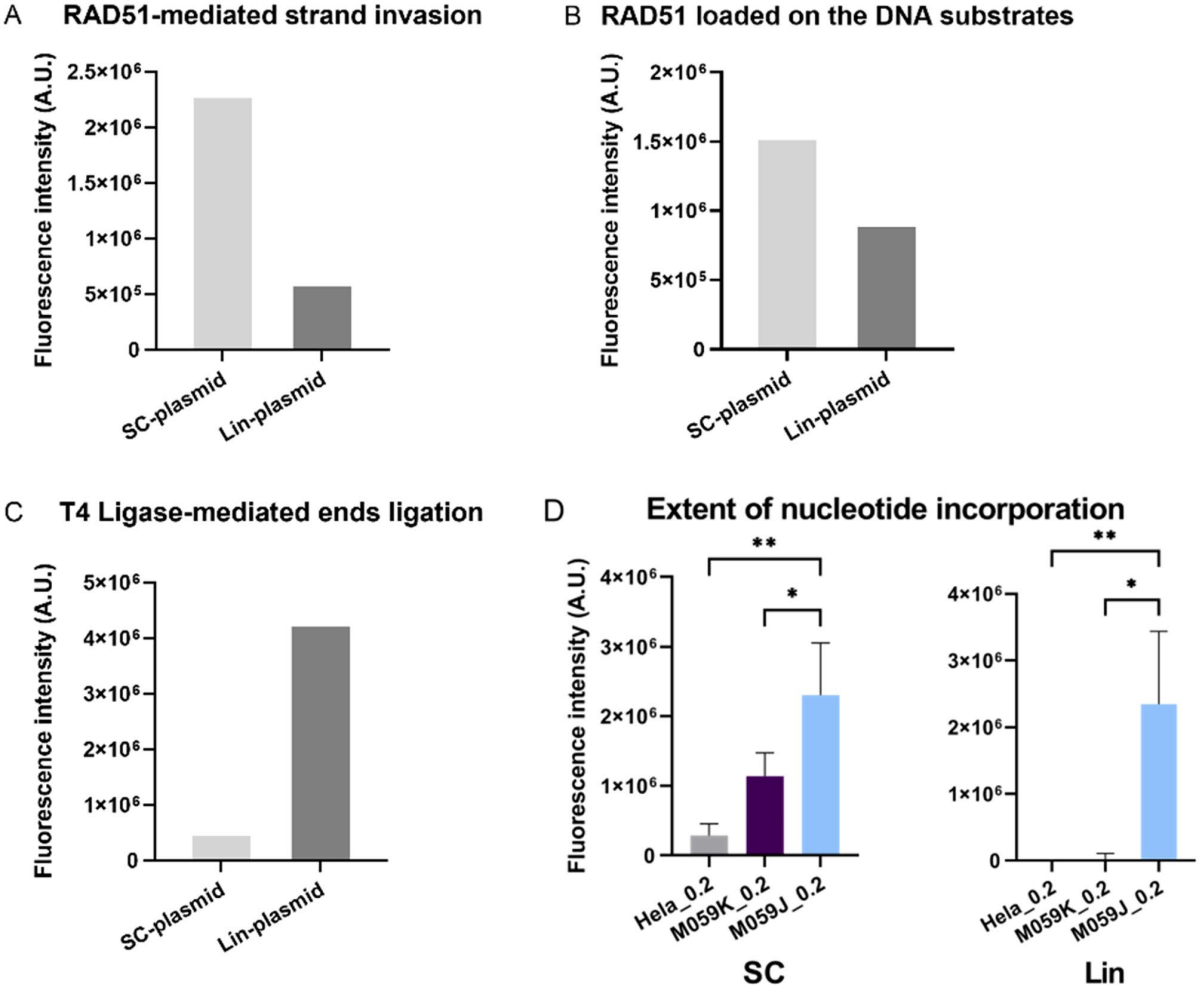
Insight into the DNA repair reaction steps on the biochip. A) RAD51-mediated strand invasion of the labeled linear plasmid (Cy3-Lin-plasmid) into the supercoiled DNA substrate (SC-plasmid) and into the linear DNA substrate (Lin-plasmid) immobilized on the biochip. RAD51-mediated strand invasion was strongly promoted on the immobilized SC-plasmid compared to the linear substrate (3.8 fold). B) Immunofluorescence detection of RAD51 loaded onto the plasmid substrates immobilized on the biochip after the repair reaction. RAD51 was more abundant on the SC-plasmid compared to the Lin-plasmid (1.7 fold). C) T4 Ligase allows the specific ligation of the Cy3-Lin-plasmid end with the complementary end of the immobilized Lin-plasmid. D) Lysate-induced single-stranded structures measuredthrough the incorporation of Cy5-dCTP by Klenow after incubation with HeLa, M059K, and M059J lysates, without dNTPs, on the two immobilized substrates (SC- and Lin-plasmids). Three independent experiments were performed (n = 3) for the three cell lines (mean ± SD are depicted). * p < 0.05, ** p < 0.01 (Anova test). *A.U.* arbitrary units, *SC* supercoiled, *Lin* linear.

Emblematic steps specific to HR and NHEJ were directly characterized on the biochip, namely strand invasion and end-joining, respectively. During HR, RAD51 binds single and double-stranded DNA and catalyzes the strand exchange between homologous DNA templates^21,22^. First, we demonstrated that RAD51 preferentially mediated the invasion of the Cy3-Lin-plasmid into the SC-plasmid. To do so, the lysates were first incubated with the Cy3-Lin-plasmid to allow the formation of a single-stranded DNA (through resection) and then, a recombinant RAD51 protein was added to the repair mix and incubated on the biochip. RAD51 effectively increased the amount of Cy3-Lin plasmid bound to the immobilized SC-plasmid by 3.8-fold compared to the amount of Cy3-Lin plasmid bound to the immobilized Lin-plasmid (Fig. 2A). Second, immunofluorescence staining of RAD51 trapped on the biochip after the repair reaction revealed that the endogenous RAD51 protein was 1.7 times more abundant on the SC-plasmid than on the Lin-plasmid (Fig. 2B). These data are consistent with RAD51 forming a nucleofilament on the resected and single strand Cy3-Lin-plasmid and invading the immobilized homologous duplex SC-plasmid. In agreement with our hypothesis, the Cy3 signal measured on the SC-plasmid substrate would be related to expected D-loop formation, a central intermediate of HR^23^.

The positive control for DSB end-joining was provided by quantifying the Cy3-Lin-plasmid bound to the substrates immobilized on the biochip after incubation with T4 DNA ligase. The fluorescent signal was detectable almost only on the immobilized Lin-plasmid (Fig. 2C). We confirmed here that ligation of Cy3-Lin-plasmid occurred specifically on the substrate containing a DSB (immobilized Lin-plasmid) and only in the presence of the ligase, as expected for NHEJ^24^. However, to further ensure that the signal observed on the biochip was due to the end ligation product, the biochip was washed several times with water (without salt) after the repair reaction. Because of the very short overlap between the two strands when NHEJ occurs, the ends ligation is necessary not to lose the ligated plasmid after the washing steps.

Another characteristic that regulates the DSB repair choice is the DNA end configuration^24^. Short or long DNA end resection is one of the fundamental steps to initiate HR, SSA, or alt-EJ^8^. To hypothesise the likely mechanism adopted by the enzymes in the lysates to repair DSB on the biochip, we characterized the extend of repair mediated DNA synthesis on the immobilized substrates through the incorporation of Cy5-dCTP into the substrates. We tested the commonly used HeLa cell line (epithelial cervix carcinoma), M059K and M059J cell lines, which have the same genetic background as they were both derived from the same malignant glioblastoma, although M059J permanently lacks the DNA-PK protein^25^ (Supplementary Fig. S1), which is involved in NHEJ. The nuclear enzymes were tested for their ability to perform neo-DNA synthesis. To this aim, we first incubated the lysates, without dNTPs, on the biochip. Then, the Klenow fragment was used to fill in any gaps created, with Cy5-dCTP. The difference between the signal obtained with lysates minus the signal obtained with the Klenow only, represents the new DNA synthesis. Note that as the lysate was removed when the Klenow was incubated on the biochip, only accessible single-strand structures will be filled in by the Klenow. Homologous strands of SC-plasmid, even if strand invasion has occurred, will be re-hybridized and will be less likely to be accessible than resected ends. Incorporation of labeled dNTP into the SC-plasmid was indeed detected for all 3 cell lines tested, to a greater extent for M059K and M059J than for HeLa cells. Incorporation of labeled dNTP into the Lin-plasmid was detected only for M059J wich is defective for DNA-PK (Fig. 2D). This latter observation supports the presence of an alternative pathway to NHEJ for M059J, measurable on the Lin-plasmid substrate, possibly associated with the presence of single-stranded DNA. We hypothesise that this could be related to alt-EJ.

### Characterization of DNA DSBs induction in three cancer cell lines following drug exposure

To get an insight into various aspects of DSBs repair mechanisms, we first characterized the presence of DSBs in three cancer cell lines before and after 48 h of treatment with a known DSB-inducer (doxorubicin), with or without DDR inhibitors (B02, NU7026 or olaparib, respectively inhibiting RAD51, DNA-PK or PARP1) through the immunofluorescent quantification of 53BP1 foci, 48 h after treatment, using automated quantification (CellInsight CX5, ThermoFisher Scientific, USA).

**Figure 3:**
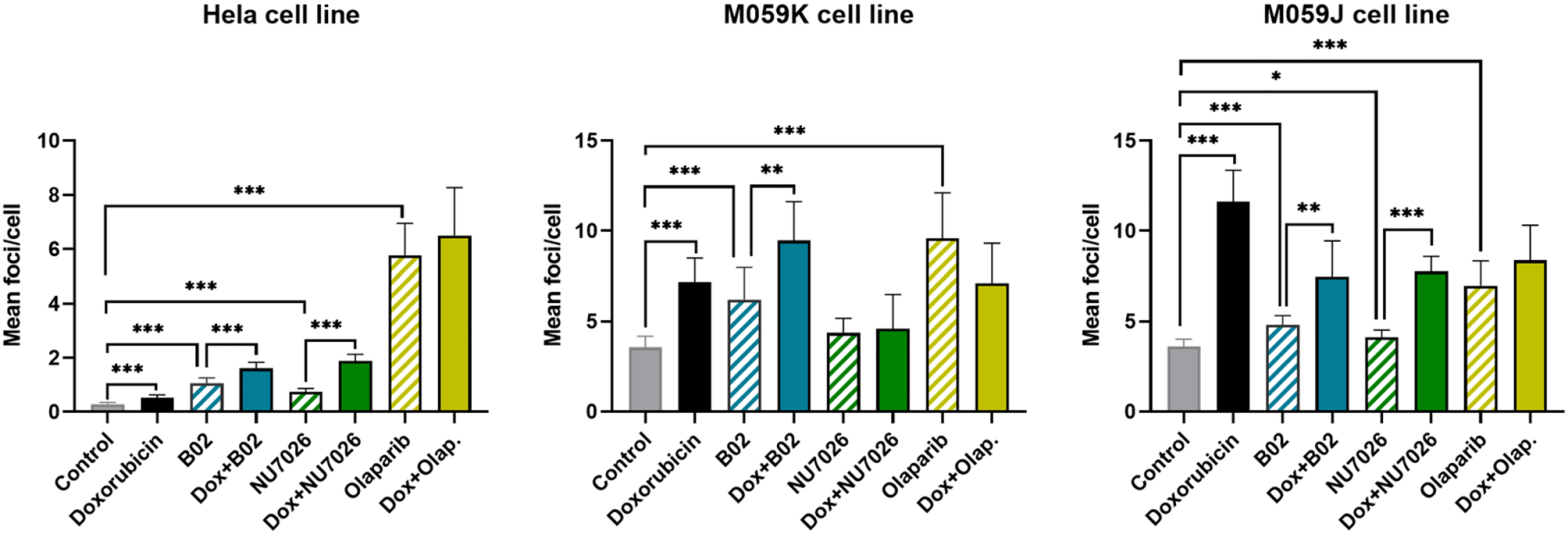
The 53BP1 foci in three cell lines after treatment with or without doxorubicin and/or DSB repair inhibitors. 53BP1 foci were measured in HeLa, M059K, and M059J cells after 48 h-treatment with doxorubicin at IC_10_ combined or not with B02, NU702, and olaparib inhibitors at IC_50_. Three independent experiments were performed (n = 3) for the three cell lines (mean ± SD are depicted). *p < 0.05, **p < 0.01, ***p < 0.001 (Wilcoxon-Mann–Whitney test). *Dox* doxorubicin, *Olap* olaparib.

53BP1 foci staining revealed a basal foci number of less than one per HeLa cell (0.3 ± 0.1 foci/cell), as compared to 3.6 foci per cell in M059J (3.6 ± 0.4 foci/cell) and M059K cells (3.6 ± 0.6 foci/cell) (Fig. 3), suggesting that DNA-PK deficiency does not impact the presence of DSBs in normal conditions. Treatment with doxorubicin (IC_10,_ according to Supplementary Figure S2) led to a significant increase in 53BP1 foci number in all cell lines (p < 0.001) (Fig. 3) thereby confirming the genotoxic effect of doxorubicin at the chosen concentration. A greater impact on 53BP1 foci was observed in the cell line lacking DNA-PK (M059J), showing that DNA-PK deficiency may impact the overall response to DSB.

Exposure to DDR inhibitors alone (IC_50_) also significantly increased the number of foci in all cell lines, except in M059K cells when treated with NU7026 (DNA-PK inhibitor). Combined treatments associating RAD51 or DNA-PK inhibition with doxorubicin, induced more damages than RAD51 or DNA-PK inhibition alone, while the numbers of 53BP1 foci following PARP1 inhibition were not significantly affected by the addition of doxorubicin (Fig. 3).

### Quantification of PARP activity following pharmacological PARP inhibition

PARP inhibitors are approved for the treatment of previously treated *BRCA*-mutant ovarian and breast cancer. PARP enzymes act as important actors in DNA single-strand break repair. In addition to inhibiting PARP catalytic activity, PARP inhibitors trap PARP proteins onto the DNA, which ultimately generates double-strand damages^26^, as we observed in Fig. 3, although the precise DSB repair mechanisms engaged by the treated cells remain unclear. It has been suggested that trapped PARP forms replication obstacles that result in the formation of DSBs in replicating cells^27^, which are repaired by HR-mediated repair pathway^28^.

**Figure 4:**
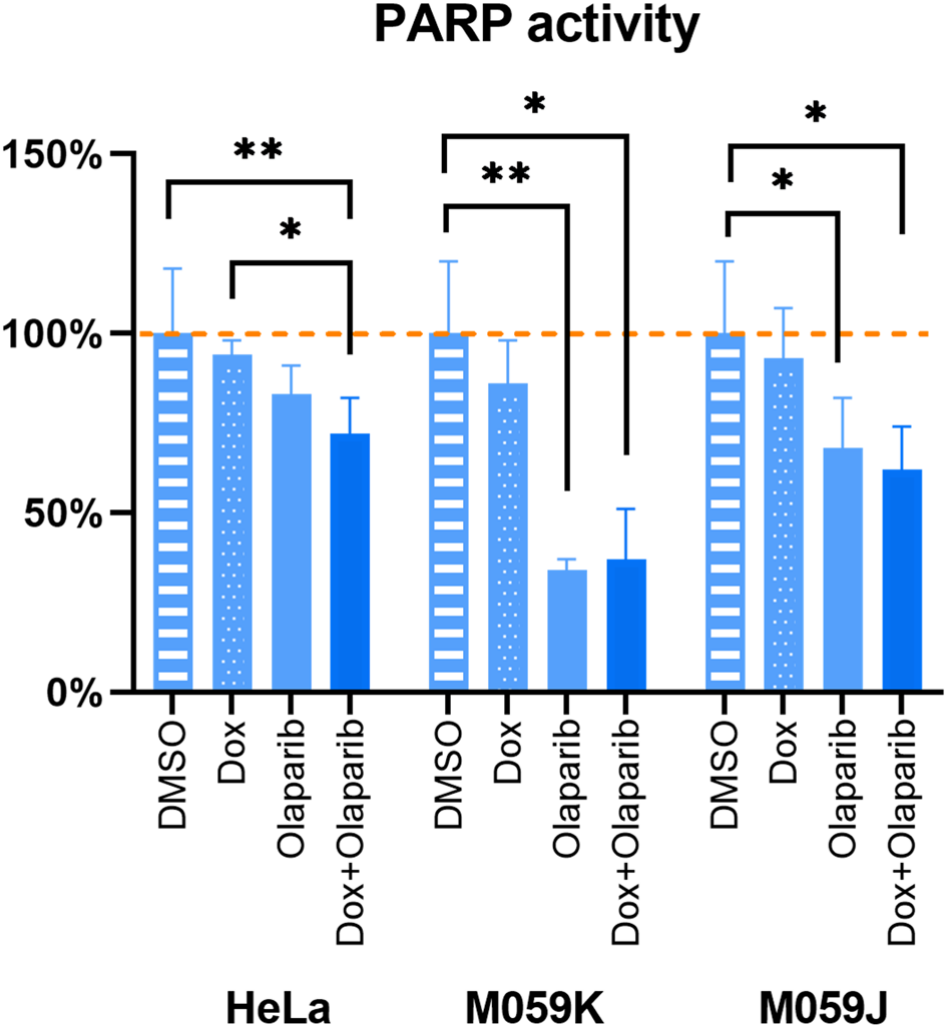
PARP activity after olaparib exposure. PARylation activity quantified with ELISA after 48 h of exposure to doxorubicin (IC_10_) combined or not with olaparib (IC_50_). Data are shown as a ratio to the control condition (DMSO only). Three independent experiments were performed (mean ± SD are depicted); *p < 0.05, **p < 0.01 (non-parametric Dunn test). Dox: doxorubicin.

Before studying the effect of olaparib on DSB repair pathways, we estimated the PARP catalytic activity of our cell lines by quantifying the NAD-dependent addition of poly(ADP-ribose) (PARylation) using an ELISA system (R&D Systems, USA). As shown in Fig. 4, olaparib (IC_50_) inhibited 17%, 32%, and 66% of the PARP activity in HeLa, M059J, and M059K cell lines, respectively. Exposure to doxorubicin alone did not alter the PARylation, neither did its combination with olaparib as compared to olaparib alone.

### NEXT-SPOT assay: functional aspects of DSB repair after exposure to DDR inhibitors alone or combined with DSB-inducer

**Figure 5:**
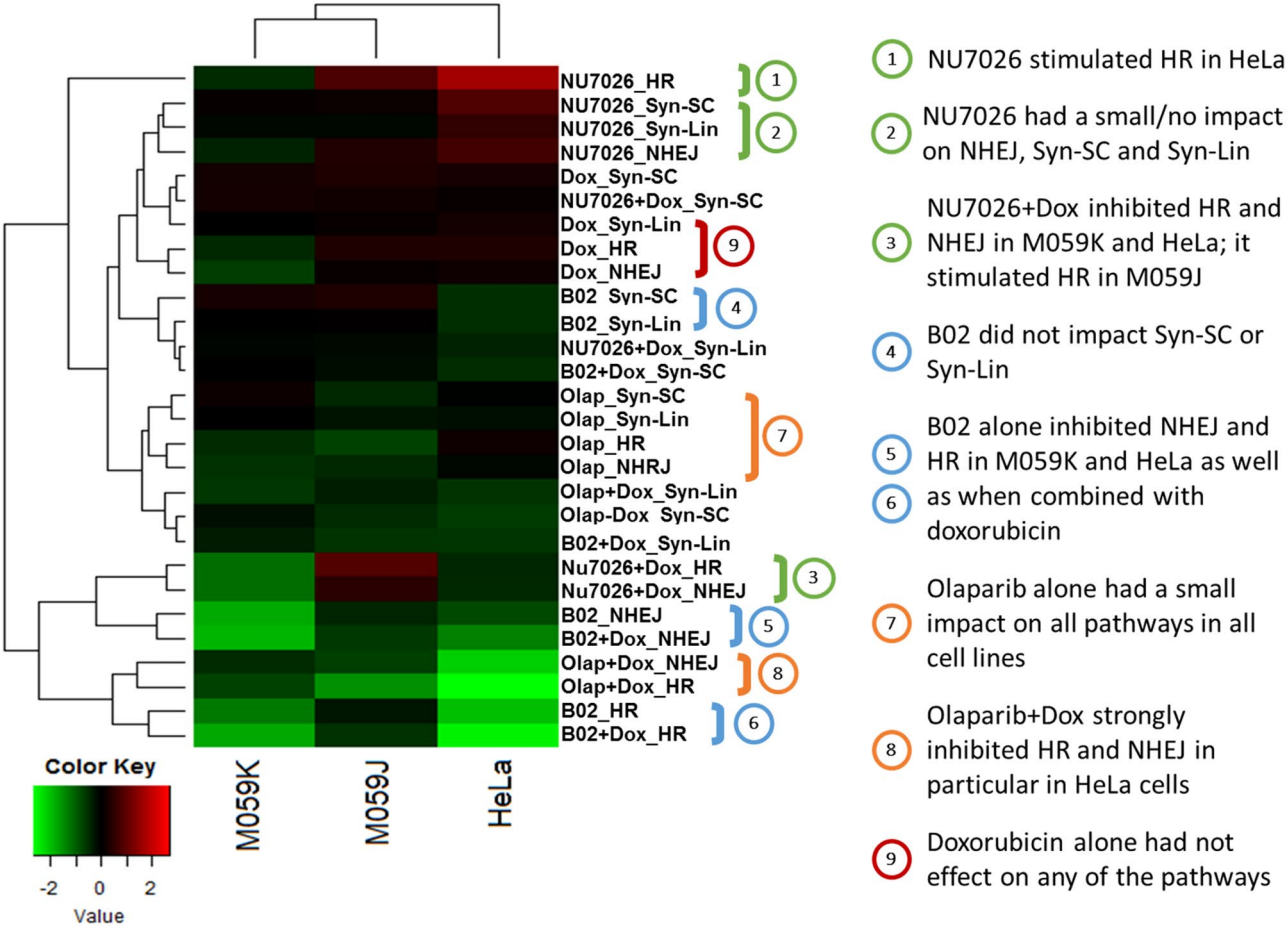
Unsupervised hierarchical clustering with Euclidean dissimilarity of DSB repair responses across treatments in three cell lines. Heatmap based on the log2 ratios of the fluorescence intensity between treated and non-treated samples (log2(T/NT)). Positive values are shaded in red and indicate a stimulation of repair activities compared to control cells, whereas negative values, colored in green, indicate an inhibition. Values around zero (in black) indicate no significant effect of the treatment. Green brackets highlight results after NU7026 exposure (groups 1, 2 and 3); blue brackets highlight results following B02 exposure (groups 4, 5, 6); orange brackets highlight results following olaparib exposure (groups 7 and 8); red bracket highlights results following doxorubicin exposure (group 9). *HR* strand invasion, *NHEJ* end-joining, *Syn-SC* DNA synthesis on SC-plasmid, *Syn-Lin* DNA synthesis on Lin-plasmid, *Dox* doxorubicin, *Olap* olaparib.

To study whether and how DDR inhibitors affect the cellular response to DNA DSBs in selected cell lines, we quantified their DSB repair following exposure to DDR inhibitors with and without doxorubicin, using the NEXT-SPOT assay. Specifically, we wanted to investigate whether DNA repair could be triggered by doxorubicin. We performed a hierarchical clustering analysis using the ratios of the signals treated/untreated (Fig. 5). Each cell line expressed a specific repair profile; however, it should be noted that those of M059K and M059J are close. In addition, the signatures were clustered mainly according to the treatment type with some notable exceptions (See below and Supplementary Fig. S3).

Pharmacological inhibition of DNA-PK (NU7026) had the most heterogeneous effects according to the genetic background of the cells: all repair activities were increased in HeLa cells, while they tended to be decreased in M059K cells, and were not affected in M059J cells (DNA-PK deficient) (Fig. 5, groups 1 and 2). When combined with doxorubicin, strand invasion and end-joining (refered in the Fig. 5 to as HR and NHEJ, respectively) were decreased in HeLa and M059K cells, while strand invasion was slightly increased in M059J cells, perhaps as a compensatory cellular response (Fig. 5, group 3).

Inhibition of RAD51 (B02) strongly inhibited HR and NHEJ pathways in DNA-PK-proficient cell lines (HeLa and M059K), regardless of the addition of doxorubicin (Fig. 5, groups 5 and 6). The effect of RAD51 inhibition was limited on the DNA synthesis activities (Syn-SC and Syn-Lin) in all cell lines (Fig. 5, group 4).

We detected a modest effect on all repair activities across the three cell lines following PARP inhibition (olaparib) (Fig. 5, group 7). However, when combined with doxorubicin, olaparib strongly inhibited HR and NHEJ pathways (Fig. 5, group 8).

Finally, doxorubicin alone had a limited effect on all activities (Fig. 5, group 9). Overall, these data suggest that DNA-PK inhibition with NU7026, and PARP inhibition with olaparib, may exert a stronger inhibitory effect on strand invasion and end-joining (HR and NHEJ) when combined with doxorubicin, while doxorubicin at the concentration used, does not modulate DSB repair response by itself.

### Reproducibility of findings with a reference electrophoresis assay

To further confirm that the findings obtained with the NEXT-SPOT assay could be reproduced using another validated method, we repeated the experiment using a previously published electrophoresis assay that estimates end-joining activities from cellular lysates^29^. Both techniques rely on the same plasmid substrates. The three main differences between the two methods lie in: (1) the reaction environment (*in solution* for the reference assay *versus* on a solid support for NEXT-SPOT), (2) the detection system to quantify repair activities (pre-readout electrophoresis for the reference test *versus* direct fluorescence readout for NEXT-SPOT), (3) the range of results (reference assay provides only end-joining quantification).

**Figure 6:**
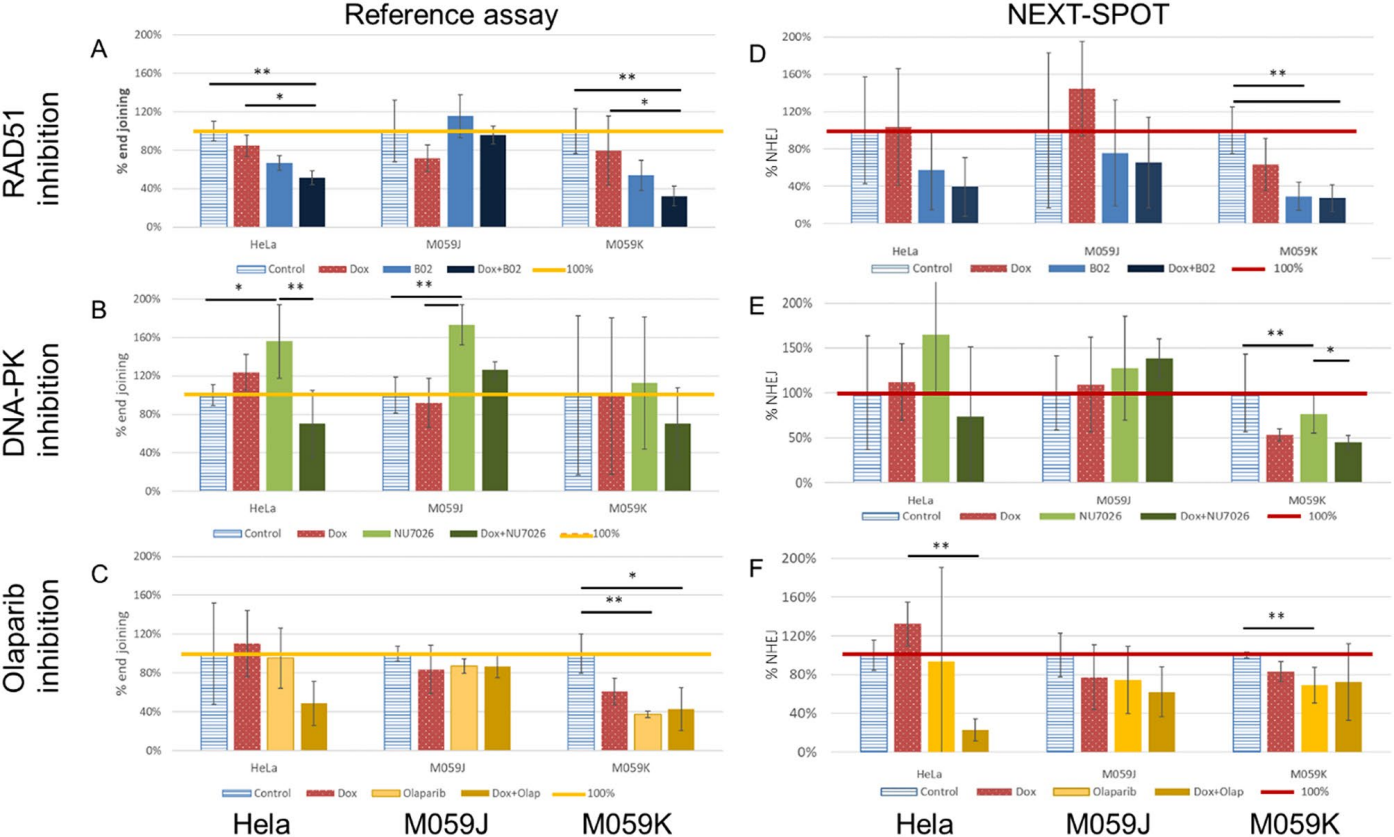
Comparison between data on end-joining obtained by reference assay and NEXT-SPOT. NHEJ activity quantified with the reference assay (**A**–**B**) and with NEXT-SPOT assay (**D**–**F**) in HeLa, M059J, and M059K nuclear extracts before and after exposure to RAD51 inhibitor (B02) (**A**,**D**), to DNA-PK inhibitor (NU7026) (**B**,**E**), to PARP inhibitor (olaparib) (**C**,**F**), combined or not with doxorubicin (red bars). Data are presented as a ratio to the control condition (non-treated cells). Three independent experiments were performed (n = 3); *p < 0.05, **p < 0.01 (non-parametric Dunn test). Error bars: standard deviation.

Both assays delivered roughly similar results regarding end-joining activity following exposure to DDR inhibitors in all three cell lines (Fig. 6). For example, the reference assay confirmed a decrease in end-joining in HeLa and M059K cells after RAD51 inhibition and PARP inhibition. Conversely, it confirmed an increase of end-joining in HeLa cells following DNA-PK inhibition, which was statistically significant only with the reference assay. Surprisingly, DNA-PK inhibition also induced a significant increase in end-joining activity in the DNA-PK-deficient cell line (M059J) when measured with the reference assay (Fig. 6B), which was also observed with NEXT-SPOT.

## Discussion

We introduced NEXT-SPOT, a novel functional assay that explores strand invasion, DNA end-joining,, and synthesis activities from the main DSB repair pathways. NEXT-SPOT tracks DNA repair by quantifying the fixation of a Cy3-Lin-plasmid and Cy5-dNTP by repair proteins contained in cell lysates onto a SC-plasmid and a Lin-plasmid immobilized on a biochip. Localization and intensity of the fluorescent signals were then automatically quantitated using a two-color biochip analysis system and correlated to mechanistic cellular behaviors.

**Figure 7:**
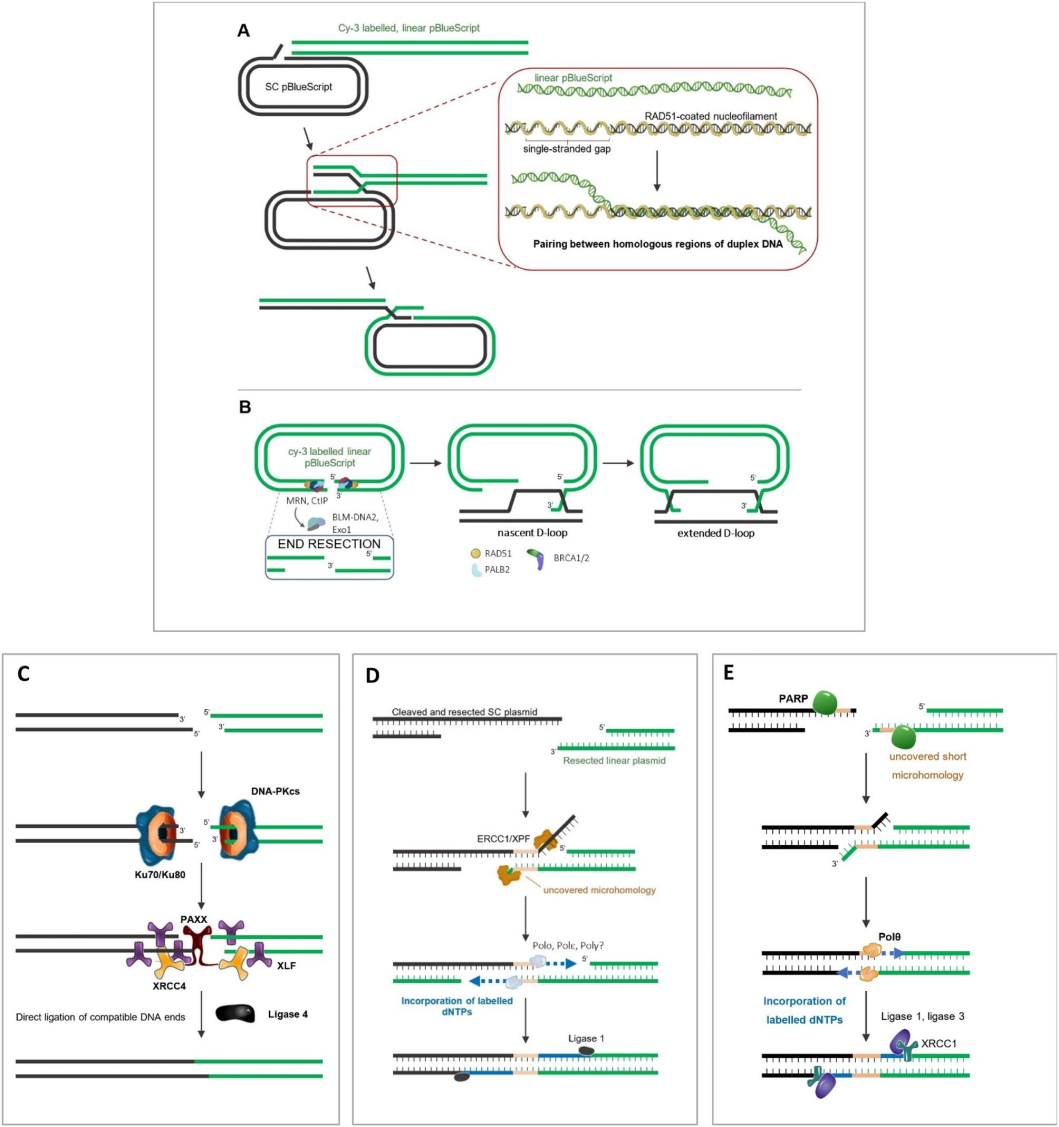
Working hypotheses for the observation of DSB repair processes. **A** HR, resection-free hypothesis. Limited cleavage of the SC-plasmid would allow the formation of single-stranded gaps triggering the recruitment of RAD51 and initiate a pairing with the Cy3-Lin-plasmid. **B** HR, resection-based hypothesis. The AflIII restriction site can be bound by the MRN complex, allowing the initiation of 5’ end resection based on the activity of BLM-DNA2 and EXO1. Strand invasion into the SC-plasmid would then be initiated following RAD51 binding on ssDNA tails, in cooperation with HR proteins BRCA1-2 and PALB2, thereby forming a recombination complex with both plasmids. The completion of HR requires DNA synthesis through several processes. **C** Ligation model of the Lin-plasmid for the observation of NHEJ-like processes. The cohesive ends present in the Lin-plasmid immobilized on the biochip and in the Cy3-Lin-plasmid are an ideal substrate for KU and DNA-PKcs proteins. This process initiates the NHEJ pathway. **D** Model of SSA-like process after SC-plasmid resection. The extensive resections occurred in the immobilized SC-plasmid after incubation with cell lysates and the presence of extensive homologies between the SC-plasmid and the Cy3-Lin-plasmid, could represent a substrate for ERCC1 and XPF which initiate the SSA pathway. Note that SDSA, which is not shown here, may be an important contributor to the DNA synthesis measured on the SC plasmid. **E** Model of alt-EJ-like process based on microhomologies. Uncovered microhomologies between the Lin-plasmid immobilized on the biochip and the Cy3-Lin-plasmid can be recognized and filled with labelled dNTPs by polθ that is involved in the alt-EJ pathway.

The conception of the biochip, the substrates used for the repair reactions, and the link between the results obtained and the specific DSB repair pathways were based on experimental designs and results from the literature. Repair proteins present within the cell lysates can reshape and excise the supercoiled and the linear plasmids offering potential substrates for the initiation of end resection, the end-joining, or the incorporation of nucleotides as part of DNA synthesis activities^5,21,30^. Figure 7 illustrates schematically the different DSB repair pathways by which the plasmids can be processed by lysates.

It has been shown that particular structures can form in supercoiled DNA and promote the initiation of recombination^31,32^. Two possible hypotheses were made based on the literature to explain the strand invasion in our system: (1) a first model was proposed based on the investigation of HR intermediates between DNA duplexes^33,34^. Following nucleolytic cleavage of the supercoiled plasmid, DNA nicks could initiate the recruitment of RAD51 to form a RAD51-ssDNA filament and allow a homologous pairing with the fluorescent linear plasmid (Fig. 7A). Then RAD51 initiates a strand exchange between the two plasmids. (2) An alternative mechanism mobilizes the MRN complex and other resection factors on the 5’ tails of the linear plasmid to initiate the formation of 3’ overhangs on each side of the restriction site (Fig. 7B). The complete homology between the linear and supercoiled plasmids would result in the formation of a displacement loop following the recruitment of RAD51, BRCA1/2, and PALB2 on the single-stranded DNA flaps^35^. The next stages involve the extended synthesis of DNA through either the double-strand break repair (DSBR) or through the synthesis dependent strand annealing (SDSA)^22^. Our results showed that the recombinase RAD51 preferentially allows the invasion of the immobilized SC-plasmid with the Cy3-Lin-plasmid (Figs. 2A), as already demonstrated by Wang et al.^36^. In addition, we demonstrated that, as expected for HR reaction, the recruitment of RAD51 is favored on the SC-plasmid compared to the Lin-plasmid (Fig. 2B). Collectively, this suggests that the supercoiled plasmid immobilized on the biochip serves as a substrate for HR-like recombination in the presence of the Cy3-labeled linear plasmid. Further evidence from BRCA2 HeLa SilenciX® models confirmed that BRCA2 silenced cells do not perform RAD51-mediated strand invasion^36^ irrespective of reaction time and protein concentration, resulting in a limited HR relative contribution compared to the mock Ctrl HeLa SilenciX® (Supplementary Fig. S5).

For its part, the Lin-plasmid immobilized on the biochip presents cohesive ends with very short 5’ overhangs (5’-CATG), an ideal substrate for KU and DNA-PK to bind DNA ends and initiate NHEJ (Fig. 7C)^24^. The KU heterodimer binds short 5’ overhangs and mobilizes DNA-PK for the recruitment of scaffold proteins and Ligase 4, which operates the ligation of plasmid ends. Furthermore, incubation with T4 DNA ligase, which has been used as a control for the last step of NHEJ^37^, results in high levels of Cy3-Lin-plasmid fixation on the immobilized Lin-plasmid (Fig. 2C), showing that the printed Lin-plasmid is a good substrate to track NHEJ-like ligation process.

Fluorescence measurement at 532 nm on the SC-plasmid can also display events attributed to the DNA pairing and recombination-associated DNA synthesis, as described by Liu et al.^38^. Following the initiation of HR, repair can be shunted to SSA, which is a faster mechanism that does not rely on strand invasion and D-loop formation, as opposed in the classical HR sub-pathways^39^; it can also be used as a backup option if the recombination process stalls or if extensive homologies are found within the resected 3’ overhangs^6^. In our case, as shown in Fig. 2D, extensive dNTPs incorporation was more likely to occur on the SC-plasmid in the presence of the lysates, than on the Lin-plasmid on which DNA ends are more readily bound to KU. The annealing between the SC-plasmid and the reshaped Cy3-Lin-plasmid could be associated with the alternative SSA pathway (Fig. 7D), which cannot be differentiated here from the RAD51-mediated strand invasion. The de novo DNA synthesis measured on the SC-plasmid after either strand invasion or annealing with the Cy3-Lin-plasmid, can be performed by polymerases involved either in the HR or SSA pathways. Because of these different possibilities, it was called DNA Synthesis on SC-plasmid (Syn-SC). The contribution of each sub-pathway could depend on the availability of core proteins, like RAD51 or RAD52^1,10^.

Finally, a limited resection would likely take place on the Lin-plasmid, due to the opposition of NHEJ factors such as KU. However, the mobilization of PARP was shown to compete with the activity of KU^40^ allowing extensive DNA synthesis associated with the reshaped Lin-plasmid (Fig. 2D). As opposed to SSA, alt-EJ does not necessarily require an extensive end resection since only short flanking microhomologies need to be uncovered^2,6^. In the case of NHEJ deficient models, the end-joining between the linear substrates can be performed through the alt-EJ pathway (Fig. 7E). In addition, based on the literature analysis^41^, the most appropriate candidate to incorporate labeled nucleotides on the Lin-plasmid is POLθ, whose activity requires the initiation of resection on the substrate plasmid. Hence, we suggest that the incorporation of Cy5-dNTPs in the Lin-plasmid immobilized on the biochip might represent the DNA synthesis involved during the alt-EJ. Our data on M059J support this hypothesis (Fig. 2D).

It is worth noting that these processes are not exclusive and can take place at the same time on the biochip.

We used a series of DNA repair inhibitors on three cellular models to obtain NEXT-SPOT signatures. We observed significant variations in the different DSB repair signatures obtained with the lysates from the cell lines exposed to doxorubicin and/or DNA repair inhibitors. For example, as expected, RAD51 inhibition by B02 profoundly decreased strand invasion activity in two cell lines (also shown for the relative pathway contribution in Supplementary Figure S4). This is consistent with the described mechanism of action of B02, which consists of its binding within the dimerization interface of the RAD51 filament, thereby preventing HR initiation^42^. Importantly, we did not observe any compensatory response in the other pathways but rather a concomitant decrease in NHEJ activity.

In addition, we made interesting observations regarding the cellular response to NU7026 exposure. NU7026 has been described to be a potent blocker of DNA-PK phosphorylation by competitive inhibition with ATP^43^, inhibiting the subsequent activation of key enzymes involved in NHEJ. Using the NEXT-SPOT assay, we noticed a significant decrease in NHEJ activity only in the M059K cell line. In HeLa cells, NU7026 boosted all four repair pathways, whereas none of them were affected in M059J cells, which are intrinsically DNA-PK-deficient. Using the reference electrophoresis assay for NHEJ detection, we confirmed a significant increase in NHEJ activity in HeLa cells, although a significant increase was also detected in M059J cells despite their DNA-PK deficiency. This could be explained by the fact that NU7026 has known off-target effects and also inhibits phosphoinositide 3-kinase (PI3K) and various PI3K-related kinases such as ATM and ATR^44^. Contrary to what one might intuitively expect, it has been shown that DNA-PK proteins are not essential for direct ligation of blunt ends^45^, which supports the fact that DNA-PK-deficient cells (M059J) or cells exposed to DNA-PK inhibitor may still perform NHEJ. Many studies rely on M059J’s deficiency to assess repair via alt-EJ, a mechanism we could observe on the biochip. In any case, our results suggest that this cell line can efficiently perform DNA end-joining, suggesting that particular attention is required when using this model.

Altogether, our data suggest that the NEXT-SPOT assay is an effective system for rapidly providing a DSB repair profile reflecting the involvement of multiple DSB repair pathways in human cells. With this comprehensive approach, NEXT-SPOT is an effective tool to support the development of DNA repair inhibitors and better understand their mechanism of action. NEXT-SPOT could be used on tumor samples as a biomarker provider to predict tumor response to a particular treatment, and also on healthy cells to predict potential side effects of treatments related to individual susceptibilities^46,47^.

## Conclusion

Repair proteins and pathways, their activation, regulation and interactions have been extensively described on their own, without a clear global view of each pathway contribution at a particular point in time. DSB repair proteins are involved in several pathways and repair pathways are intertwined, which makes it difficult to state on whether or not a mechanism is deeply affected by a molecular alteration or a pharmacological inhibition. Multiple observations and mechanistic modeling support the statement that the ability of cells to choose selectively between the different DSB repair pathways is an oversimplification. There is a need to better understand the orchestration and the dynamic of repair pathways, as well as the real impact on the different types of damage repair at the functional level, using more comprehensive systems. The functional multiplex approach offers a less reductionist view of DSB repair and reveals the global interplay between pathways.

## Materials and methods

### Preparation of nuclear protein lysates

Nuclear protein lysates were prepared by incubating PBS-rinsed cellular pellets on ice for 20 min in a hypotonic buffer (10 mM HEPES–KOH, 10 mM KCl, 1.5 mM MgCl_2_, 0.5 mM DTT, 103 µM PMSF, 0.02% Triton X-100). Then this buffer was discarded after centrifugation at 2.300 g and a hypertonic buffer (10 mM HEPES–KOH, 0.2 mM EDTA-NaOH, 400 mM KCl, 1.5 mM MgCl_2_, 0.5 mM DTT, 103 µM PMSF, 0.7X protease inhibitor, 25% glycerol) was used to resuspend the nuclei. Nuclei lysis was facilitated by two freezing–thawing cycles and a final centrifugation at 16,000×*g* for 10 min. Protein concentration in the cell lysates was determined using the MicroBC Assay (Interchim, France), according to the manufacturer’s instructions. Lysates were then stored at -80°.

### Preparation of SC-plasmid and of DSB-plasmid substrates

The supercoiled pBlueScript plasmid (SC-plasmid; Stratagene) was prepared as described in Millau et al.^48^. Linear plasmid (Lin-plasmid) was obtained by digestion of pBlueScript plasmid using the restriction enzyme *Afl*III following the manufacturer protocol (New England Biolabs). Then, the Lin-plasmid was precipitated with isopropanol/sodium acetate and suspended in molecular biological-grade water at the desired concentration.

The Cy3-labeled Lin-plasmid (Cy3-Lin-plasmid) was obtained using the Label IT® Tracker™ Kit (Mirus) following the supplier instructions. Briefly, 100 µg of Lin-plasmid were incubated with 50 µL of Label IT® Reagent and 100 µL of Labeling Buffer 3.1 for 2 h at 37 °C. Then, the Cy3-Lin-plasmid was precipitated in Ethanol/NaCl, diluted in molecular biological-grade water and stored at − 80 °C.

Cy3-Lin-plasmids were quantified using the NanoDrop™ spectrophotometer; labeling ratio was calculated based on the manufacturer’s instructions. Estimates were on average of approximately one label every 200 base pairs.

### Preparation of the DSB repair biochips

SC-plasmid and Lin-plasmid were immobilized on coated glass slides (Nexterion® H, Schott) at specific positions using an ultra-low volume dispensing system (SciFlexarrayer, Scienion Germany). Fourteen identical pads were formed, each containing 4 spots of unlabeled plasmids (2 SC-plasmids and 2 Lin-plasmids). The process described by the slide manufacturer was followed for plasmids immobilization and support inactivation. The slides were stored under vacuum at − 20 °C.

### DSB repair profiling using NEXT-SPOT assay

An HybriWell™ Sealing System (Grace Bio-Labs) was applied on the biochip to form 14 identical reaction chambers. Each lysate was tested at 2 different final protein concentrations. To that aim, a mixture containing (i) 2 ng/µL of Cy3-labeled linear DSB plasmid (Cy3-Lin-plasmid), (ii) 0.25 µM of biotin-dCTP, 0.25 µM of the 3 others non labeled dNTPs, (iii) 80 mM KCl, 20 mM Tris–HCl pH 7.5, 10 mM MgCl_2_, 2 mM DTT, 0.1 mg/mL BSA, 1 mM ATP, 0.05 mg/mL Creatin Phosphokinase, 10 mM Phosphocreatine in a total reaction volume of 12 µL per well was prepared. The repair reaction was carried out during 1 h at 30 °C. The slide was then rinsed twice with Milli-Q water. The biotin-dCTP was subsequently labeled by incubation with 0.1 µg/mL Streptavidin-Cy5 solution, for 30 min at 30 °C. Then, the slide was rinsed twice with Milli-Q water and dried out. Each sample was tested on two pads (technical replicates). Alternatively, instead using biotin-dCTP, Cy5-dCTP was used as direct label.

### Fluorescence quantification and data analysis

Fluorescent signals were quantified using a scanner (Innoscan 710AL from Innopsys, and the Mapix software) at two wavelengths, 532 nm (Cy3) and 635 nm (Cy5). Results, calculated as the mean of the fluorescence intensity of 4 replicata, were expressed as Fluorescence Intensity (Arbitrary Units). Each sample was characterized by 4 values referred as strand invasion HR-like, end-joining NHEJ-mediated, DNA Synthesis on SC-plasmid (Syn-SC) and DNA Synthesis on Lin-plasmid (Syn-Lin), for each protein concentration tested.

### Statistical analysis for DNA repair experiments

Triplicates were prepared for each cellular experimental condition. The means were then calculated. NEXT-SPOT data were presented either as treated-to-untreated ratio, or as relative pathway contributions (Supplementary Figs. S4, S5). Unsupervised hierarchical clustering was used to visualize the associations between treatment effects in the different cell lines, using the “pheatmap” and “pvclust” R packages. Plotted data corresponded to the base 2 logarithm of the ratio between NEXT-SPOT data from treated and untreated cells. Clustering was based on Euclidean distance, which regroups profiles with both similar intensity levels and covariation. This classification considers both the co-regulation of repair pathways and intensity level. pvclust provides two types of p-values, bootstrap probability and approximately unbiased p-value. The latter has superiority in bias over the value calculated by the ordinary bootstrap resampling^49^ and clusters with approximately unbiased p-value above 95% were considered significant.

All statistical analyses were performed using RStudio v.1.4.1103 based on R v.4.0.3 (www.cran.r-project.org) and GraphPad Prisme 9. In case groups were large enough, pairwise comparisons were run using Mann–Whitney–Wilcoxon rank sum tests with Bonferroni correction. Otherwise, we performed pairwise Anova and Dunn tests to identify significantly different groups; the standard significance level for this test is a *p*-value equal to 0.05.

### 53BP1 and RAD51 immunofluorescence analyses in cells and on the biochip, respectively

Similar protocols were followed for the immunofluorescence analysis of cells components or for proteins captured on the DNA substrates immobilized on the biochip. Cells were fixed for 30 min at room temperature with 4% formaldehyde solution after 48 h of treatment; this step was omited for the biochip. The detection of proteins trapped on the biochip was performed after the repair reaction. Before proceeding with the protein detection, cells and biochips were treated for 15 min with PBS containing 3% BSA and 0.2% Triton X-100. Cells were incubated for 1 h with anti-53BP1 primary antibody (1:1,000, Abnova Corporation, Taiwan) diluted in PBS containing 3% BSA and 0.02% sodium azide. Then, anti-rabbit secondary antibody (1:2,000, Atto 488, Sigma-Aldrich, USA) diluted in PBS-3% BSA was added to each well and incubated for 1 h in the dark. Cell nuclei were stained with 0.3 µg/mL Hoechst 33342 (Sigma-Aldrich, USA). The biochips were incubated with anti-RAD51 primary antibody (1:2,000, AbCam, USA) and anti-rabbit secondary antibody (1:5000, 635 AlexaFluor, USA).

53BP1 foci were counted in 500 cells per each tested condition using an automated high-content screening platform (CellInsight CX5, ThermoFisher Scientific, USA). Exported parameters included mean foci total fluorescence and area per cell, as well as the average foci number per cell.

Fluorescence associated with the RAD51 protein was quantified using a microarray scanner (Innopsys Innoscan 710AL, and Mapix software).

### Investigation of single-stranded structures in the substrates immobilized on the biochip

The aim of this experiment was to evaluate the presence of single-stranded structures created during the repair reaction. To quantify the initial presence of single-stranded structures in the immobilized substrates, the biochip was incubated with the Klenow fragment of DNA polymerase I (0.032 U/µL in the supplied 1X buffer (New England Biolabs), 20 min at 37 °C) in the presence of Cy5-dCTP and of the other dNTPs. The enzyme filled the gaps or the protruding ends of the SC-plasmid and the Lin-plasmid (basal single-stranded structures). Then, the repair reaction was performed in the presence of the lysate, Cy3-Lin-plasmid but without dNTPs, and later the Klenow was used to reveal the new single-stranded structures created by the repair enzymes present in the lysate. The signal attributed to the new DNA synthesis corresponded to the difference between the signal obtained with lysates and Klenow (total single-stranded structures) minus the signal obtained with the Klenow only (basal single-stranded structures). The ability of HeLa, M059K and M059J lysates to create single stranded structures accessible to the Klenow enzyme were thus investigated.

### RAD51-mediated strand invasion on the plasmid substrates immobilized the biochip

The ability of exogenous RAD51 to specificaly promote strand invasion and boost the signal related to Cy3-Lin-plasmid on the SC-plasmid was evaluated. As a first step, to allow resection and then the nucleofilament to be formed, the Cy3-Lin-plasmid (2 ng/µL) was incubated 15 min at 37 °C with the lysates at 0.4 mg/mL, in the repair buffer containing ATP and dNTPs.

Next, recombinant human RAD51 protein (Abcam, USA) was added to the mixture at 0.4 µM final concentration and incubated on the biochip for 1 h at 30 °C. The slide was then rinsed twice with Milli-Q water and dried out. The strand invasion mediated by exogenous RAD51 was the resultant of the signal obtained with the combination of RAD51 and lysate minus the signal obtained with lysate alone. It allowed to compare the ability of RAD51 protein to promote the Cy3-Lin-plasmid invasion on both the SC-plasmid and on the Lin-plasmid immobilized on the biochip, and thus check HR-related strand invasion on the SC-plasmid.

### Quantification of PARP activity

PARP activity was measured in nuclear lysates using a ELISA system (R&D Systems, USA) which tracks the NAD-dependent addition of poly(ADP-ribose). Lysates were diluted in their original buffer to a protein concentration of 1 µg/µL. Serial dilutions were made in the supplied PAR Standars solution up to 2 ng/µL and 25 µL of diluted lysates were dispensed onto a histone-coated 96-well plate. After addition of 25 µL of reaction mix containing activated DNA and 2 mM NAD into each well, the plates were incubated at room temperature for 30 min. Following 2 rinses in PBS-0.1% Triton X-100 and 2 rinses in PBS, 1:1000 anti-PAR antibody was added for a 30-min incubation. After rinsing, 1:1000 anti-mouse IgG-HRP conjugate was dispensed and incubated for 30 min. The wells were rinsed again, PeroxyGlow™ solutions were added and the plate was taken to the automated reader (SpectraMax® iD3, Molecular Devices, USA) for chemiluminescent measurement. Results were expressed in PARP mU/µl based on internal calibration standards. When investigating cellular treatments, data was normalized to the untreated controls.

### Reference method: end-joining in solution and electrophoretic analysis

Triplicates were prepared; normalized as presented in captions, usually as a ratio to the control condition. In this case, plotted error bars represent standard deviation (SD) adjusted by the ratio between the plotted value and the control. The reference method was set up based on published protocols for classical cell-free NHEJ assays^29^. More specifically, the electrophoretic assay explores ligation activities from the NHEJ pathway by tracking the oligomerization of the Lin-plasmid digested with *Afl*III enzyme. Basically, as for NEXT-SPOT, the SC-plasmid was linearized with *Afl*III and incubated with cell lysates diluted in a repair mix. The repair mix contained 2 mM HEPES–KOH, 40 µM EDTA-NaOH, 10 mM MgCl_2_, 80 mM KCl, 1 mM ATP, 50 µg/mL Creatine phosphokinase, 10 mM Phosphocreatine, 2 mM DTT, 0.1 mg/mL BSA, 0.25 µM of dNTPs and 5 ng/µL of Cy3-Lin-plasmid. Tubes were incubated for 1 h at 30 °C to allow plasmid ligation. Proteinase K (PK; Sigma-Aldrich, USA) was then added to a final concentration of 0.5 mg/mL and incubated for 30 min at 37 °C. Ligation products were separated by 3 h electrophoresis run at 120 V in a 0.8% agarose gel stained with 1 µg/mL ethidium bromide. The signal intensity of each band obtained with the imager (ChemiDoc XRS+, Bio-Rad, USA) was expressed as a ratio to the total intensity measured in the lane; repair efficcacy was expressed as the total percentage of ligated plasmid bands, as opposed to remaining non-ligated plasmids.

## Data Availability

The data sets generated and/or analyzed during the current study are available from the corresponding author on reasonable request.

## References

[1] Scully R, Panday A, Elango R, Willis NA (2019). DNA double-strand break repair-pathway choice in somatic mammalian cells. Nat. Rev. Mol. Cell Biol..

[2] Mladenov E, Magin S, Soni A, Iliakis G (2016). DNA double-strand-break repair in higher eukaryotes and its role in genomic instability and cancer: Cell cycle and proliferation-dependent regulation. Semin. Cancer Biol..

[3] Wright WD, Shah SS, Heyer W-D (2018). Homologous recombination and the repair of DNA double-strand breaks. J. Biol. Chem..

[4] Li X, Heyer W-D (2008). Homologous recombination in DNA repair and DNA damage tolerance. Cell Res..

[5] Mimitou EP, Symington LS (2009). DNA end resection: Many nucleases make light work. DNA Repair (Amst)..

[6] Bhargava R, Onyango DO, Stark JM (2016). Regulation of Single Strand Annealing and its role in genome maintenance Chromosomal break repair by the Single Strand Annealing (SSA) pathway. Trends Genet..

[7] Chang HHY, Pannunzio NR, Adachi N, Lieber MR (2017). Non-homologous DNA end joining and alternative pathways to double-strand break repair. Nat. Rev. Mol. Cell Biol..

[8] Zhao B, Rothenberg E, Ramsden DA, Lieber MR (2020). The molecular basis and disease relevance of non-homologous DNA end joining. Nat. Rev. Mol. Cell Biol..

[9] Meyer D, Fu BXH, Heyer W-D (2015). DNA polymerases δ and λ cooperate in repairing double-strand breaks by microhomology-mediated end-joining in Saccharomyces cerevisiae. Proc. Natl. Acad. Sci. USA.

[10] Ceccaldi R, Rondinelli B, D’andrea AD (2016). Repair pathway choices and consequences at the double- strand break mechanisms of DNA DSB Repair. Trends Biochem. Sci..

[11] Nickoloff JA, Jones D, Lee SH, Williamson EA, Hromas R (2017). Drugging the Cancers Addicted to DNA Repair. J. Natl. Cancer Inst..

[12] Ingram SP (2019). Mechanistic modelling supports entwined rather than exclusively competitive DNA double-strand break repair pathway. Sci. Rep..

[13] Xiao Y (2021). Comprehensive analysis of DNA damage repair deficiency in 10,284 pan-cancer study. Ann. Transl. Med..

[14] Jiang M (2021). Alterations of DNA damage response pathway: Biomarker and therapeutic strategy for cancer immunotherapy. Acta Pharm. Sin. B.

[15] Chabanon RM (2021). Targeting the DNA damage response in immuno-oncology: Developments and opportunities. Nat. Rev. Cancer.

[16] Pilié PG, Tang C, Mills GB, Yap TA (2019). State-of-the-art strategies for targeting the DNA damage response in cancer. Nat. Rev. Clin. Oncol..

[17] Pollard JM, Gatti RA (2009). Clinical radiation sensitivity With DNA repair disorders: An overview. Int. J. Radiat. Oncol..

[18] Yang L (2020). Targeting Cancer Stem Cell Pathways for Cancer Therapy. Signal Transduction and Targeted Therapy.

[19] Vítor AC, Huertas P, Legube G, de Almeida SF (2020). Studying DNA double-strand break repair: An ever-growing toolbox. Front. Mol. Biosci..

[20] Tatin X, Muggiolu G, Sauvaigo S, Breton J (2021). Evaluation of DNA double-strand break repair capacity in human cells: Critical overview of current functional methods. Mutat. Res. Mutat. Res..

[21] Budke B, Chan YL, Bishop DK, Connell PP (2013). Real-time solution measurement of RAD51- and RecA-mediated strand assimilation without background annealing. Nucleic Acids Res..

[22] Huselid E, Bunting SF (2020). The regulation of homologous recombination by helicases. Genes.

[23] Moldovan GL (2012). Inhibition of homologous recombination by the PCNA-interacting protein PARI. Mol. Cell.

[24] Chang HHY (2016). Different DNA end configurations dictate which NHEJ components are most important for joining efficiency. J. Biol. Chem..

[25] Anderson CW, Dunn JJ, Freimuth PI, Galloway AM, Allalunis-Turner MJ (2001). Frameshift mutation in PRKDC, the gene for DNA-PKcs, in the DNA repair-defective, human, glioma-derived cell line M059J. Radiat. Res..

[26] Lord CJ, Ashworth A (2017). PARP inhibitors: The first synthetic lethal targeted therapy Europe PMC funders group. Science.

[27] Bochum S, Berger S, Martens UM (2018). Olaparib. Recent Results Cancer Res..

[28] Wang H, Zhang S, Song L, Qu M, Zou Z (2020). Synergistic lethality between PARP-trapping and alantolactone-induced oxidative DNA damage in homologous recombination-proficient cancer cells. Oncogene.

[29] Sharma, S. & Raghavan, S. C. Nonhomologous DNA end joining in cell-free extracts. *J. Nucleic Acids* **2010**, 1–11 (2010). 10.4061/2010/389129 PMC2945661 20936167

[30] Smith-Ravin J, Jeggo PA (1989). Use of damaged plasmid to study DNA repair in X-ray sensitive (Xrs) strains of Chinese hamster ovary (CHO) cells. Int. J. Radiat. Biol..

[31] Frank-Kamenetskii MD, Mirkin SM (1995). Triplex DNA structures. Annu. Rev. Biochem..

[32] Rooney SM, Moore PD (1995). Antiparallel, intramolecular triplex DNA stimulates homologous recombination in human cells. Proc. Natl. Acad. Sci. U. S. A..

[33] West SC, Howard-Flanders P (1984). Duplex-duplex interactions catalyzed by recA protein allow strand exchanges to pass double-strand breaks in DNA. Cell.

[34] Lopez B, Rousset S, Coppey J (1987). Homologous recombination intermediates between two duplex DNA catalysed by human cell extracts. Nucleic Acids Res..

[35] Smirnov A (2020). DNA barcoding reveals that injected transgenes are predominantly processed by homologous recombination in mouse zygote. Nucleic Acids Res..

[36] Wang CX, Jimenez-Sainz J, Jensen RB, Mazin AV (2019). The Post-Synaptic Function of Brca2. Sci. Rep..

[37] Labhart P (1999). Nonhomologous DNA end joining in cell-free systems. Eur. J. Biochem..

[38] Liu J, Sneeden J, Heyer WD (2011). In vitro assays for DNA pairing and recombination-associated DNA synthesis. Methods Mol. Biol..

[39] Li J (2019). Pathways and assays for DNA double-strand break repair by homologous recombination. Acta Biochim. Biophys. Sin. (Shanghai).

[40] Wang M (2006). PARP-1 and Ku compete for repair of DNA double strand breaks by distinct NHEJ pathways. Nucleic Acids Res..

[41] He P, Yang W (2018). Template and primer requirements for DNA Pol θ-mediated end joining. Proc. Natl. Acad. Sci. U. S. A..

[42] Shkundina, I. S., Gall, A. A., Dick, A., Cocklin, S. & Mazin, A. V. New rad51 inhibitors to target homologous recombination in human cells. *Genes 12*, 920 (2021). 10.3390/genes12060920 PMC8235719 34208492

[43] Veuger SJ, Curtin NJ, Richardson CJ, Smith GCM, Durkacz BW (2003). Radiosensitization and DNA repair inhibition by the combined use of novel inhibitors of DNA-dependent protein kinase and poly(ADP-ribose) polymerase-1. Cancer Res..

[44] Harnor SJ, Brennan A, Cano C (2017). Targeting DNA-dependent protein kinase for cancer therapy. ChemMedChem.

[45] Crowe JL (2018). Kinase-dependent structural role of DNA-PKcs during immunoglobulin class switch recombination. Proc. Natl. Acad. Sci. U. S. A..

[46] van den Boogaard WMC, Komninos DSJ, Vermeij WP (2022). Chemotherapy side-effects: Not all DNA damage is equal. Cancers (Basel).

[47] Van Oorschot B (2017). Prostate cancer patients with late radiation toxicity exhibit reduced expression of genes involved in DNA double-strand break repair and homologous recombination. Cancer Res..

[48] Millau J-F (2008). A microarray to measure repair of damaged plasmids by cell lysates. Lab Chip.

[49] Suzuki R, Shimodaira H (2006). Pvclust: An R package for assessing the uncertainty in hierarchical clustering. Bioinformatics.

